# Risk-stratified association between dilute povidone-iodine lavage and SSI/PJI after primary total hip arthroplasty

**DOI:** 10.1051/sicotj/2026054

**Published:** 2026-10-15

**Authors:** Daisuke Inoue, Tamon Kabata, Yu Yanagi, Musashi Ima, Takahiro Iyobe, Naoya Fujimaru, Keisuke Sano, Rin Maeda, Taisuke Yamamoto, Satoru Demura

**Affiliations:** Department of Orthopedic Surgery, Graduate School of Medical Science, Kanazawa University Kanazawa Ishikawa Japan

**Keywords:** Total hip arthroplasty, Diluted povidone-iodine irrigation, Periprosthetic joint infection, Surgical site infection, Patient risk

## Abstract

*Introduction*: Unresolved issue remains regarding which patient populations benefit from diluted povidone-iodine (PI) irrigation for preventing surgical-site infection (SSI)/periprosthetic joint infection (PJI) after primary total hip arthroplasty (THA). We aimed to clarify the clinical effect of diluted PI lavage on SSI/PJI incidence in risk-stratified patients undergoing primary THA. *Methods*: This retrospective, single-center study included 1,128 patients with primary THA for osteoarthritis. We classified all enrolled patients into three groups according to the American Society of Anesthesiologists Physical Status (ASA-PS) 1, 2, and 3 groups. We compared the incidence rate of SSI/PJI between two subgroups in each group: patients who received diluted PI and those who did not. Odds ratios (ORs) for SSI/PJI were estimated for PI irrigation. *Results*: Eleven infections occurred overall (0.9%): five superficial SSIs (0.4%) and six early PJIs (0.5%). No infections occurred among patients in the ASA-PS 1 group. In the ASA-PS 2 group, eight SSI/PJI events were observed (0.8%), and in ASA-PS 3, three SSI/PJI events occurred (3.3%). In the ASA-PS 2 group, PI use was associated with a lower infection rate (*P* = 0.04), whereas no significant difference was detected in ASA-PS 3 (*P* = 0.55). OR revealed that diluted PI caused a significant reduction in SSI/PJI (OR, 0.23; *P* = 0.03) in the ASA-PS 2 group. *Discussion*: In this cohort, dilute PI lavage was associated with fewer SSI/PJI events among patients with ASA-PS class 2 who underwent primary THA; however, a clear benefit was not demonstrated in patients with ASA-PS class 3. Additional intraoperative measures may be required for higher-risk patients, such as those with ASA-PS class 3.

## Introduction

Primary total hip arthroplasty (THA) reliably alleviates pain and restores function in patients with advanced hip osteoarthritis [1]. Nonetheless, surgical-site infection (SSI) and periprosthetic joint infection (PJI) remain uncommon yet serious complications that threaten outcomes and increase healthcare burden [2].

During the pathogenesis of PJI, initial bacterial attachment to the metal surface is followed by production of an extracellular polymeric substance and biofilm formation, which substantially limits therapeutic efficacy and often necessitates implant removal [3, 4]. Hence, intraoperative bacterial attachment to the metal surface should be prevented through various preoperative and intraoperative preventive strategies for SSI/PJI. Intraoperative diluted povidone-iodine (PI) irrigation is one of the procedures used to prevent SSI/PJI. International guidelines and recent systematic reviews have highlighted the favorable outcomes of intraoperative diluted PI irrigation in preventing SSI/PJI after orthopedic surgery [5–7]. Although previous papers indicated that American Society of Anesthesiologists Physical Status (ASA-PS) influences the incidence of SSI/PJI, unresolved issues remain regarding which patient populations benefit from diluted PI irrigation for preventing SSI/PJI [8, 9].

Herein, we examined whether the clinical effect of intraoperative dilute PI lavage differs across patient risk stratification in primary THA, focusing on ASA-PS classes.

## Materials and methods

The institutional review board approved this study protocol. We performed a single-center, retrospective review of consecutive patients who underwent primary THA for hip osteoarthritis between January 1, 2008, and December 31, 2024. Exclusion criteria were simultaneous bilateral THA, THA with femoral shortening osteotomy, and follow-up shorter than 3 months.

For preoperative skin preparation, the skin from the pelvis to the foot on the operative side was disinfected using an alcohol-containing chlorhexidine solution. PI was applied to the sterilely prepped skin before draping. After draping, a PI-impregnated incision drape was positioned over the surgical site before the incision was made. For antibiotic prophylaxis, first- or second-generation cephalosporins (cefazolin or cefmetazole) were administered within 24 h postoperatively. During surgery, 0.2% diluted PI irrigation was used from January 1, 2013, whereas saline was used for intraoperative irrigation before 2013. Irrigation was generally performed every 30 min after the start of surgery using a pulsatile lavage system. In particular, irrigation was performed before acetabular and femoral implant insertion and before wound closure. Diluted PI was left in the wound for 3 min and then rinsed with saline. Finally, antimicrobial-coated sutures were generally used for wound closure.

Patient demographic data were manually obtained from the medical electronic database. These included age, sex, body mass index (BMI), ASA-PS, age-adjusted Charlson comorbidity index (CCI), diabetes mellitus (DM), and preoperative glycated hemoglobin (HbA1c) in patients who had DM. In the case of preoperative HbA1c levels, the latest data within 1 month of the planned surgery were used.

### Assessment

All candidates were screened for SSI and early PJI (≤90 days) using the Centers for Disease Control and Prevention criteria for SSI and International Consensus Meeting criteria for PJI [10, 11]. The cohort was stratified into ASA-PS 1, 2, and 3. Within each stratum, we compared infection rates between the PI and non-PI subgroups because we aimed to clarify the clinical effect of patient risk stratification on SSI/PJI incidence associated with diluted PI lavage in primary THA. Finally, odds ratios (ORs) for SSI/PJI were estimated for PI irrigation in the ASA-PS 2 group.

### Data analyses

Statistical computations were performed with EZR, an R-based graphical environment (Jichi Medical University, Saitama, Japan) [12]. Continuous outcomes (mean age, BMI, preoperative HbA1c) were compared using the Mann–Whitney U test; categorical outcomes (sex, age-adjusted CCI, rate of DM, and rate of SSI/PJI) were compared using Fisher’s exact test. Fisher’s exact test was applied to calculate the OR for SSI/PJI in the ASA-PS 2 group receiving diluted PI irrigation. Significance was defined as a two-tailed *P* < 0.05.

## Results

A total of 1374 primary THAs were identified. Exclusions were applied to 100 bilateral simultaneous operations (200 hips), 39 THAs combined with femoral osteotomy, and 7 cases lacking ≥3 months of follow-up, yielding 1128 eligible cases. Among these, 11 hips (0.9%) experienced SSI/PJI, comprising 5 SSIs (0.4%) and 6 early PJIs (0.5%) (Figure 1).

**Figure 1:**
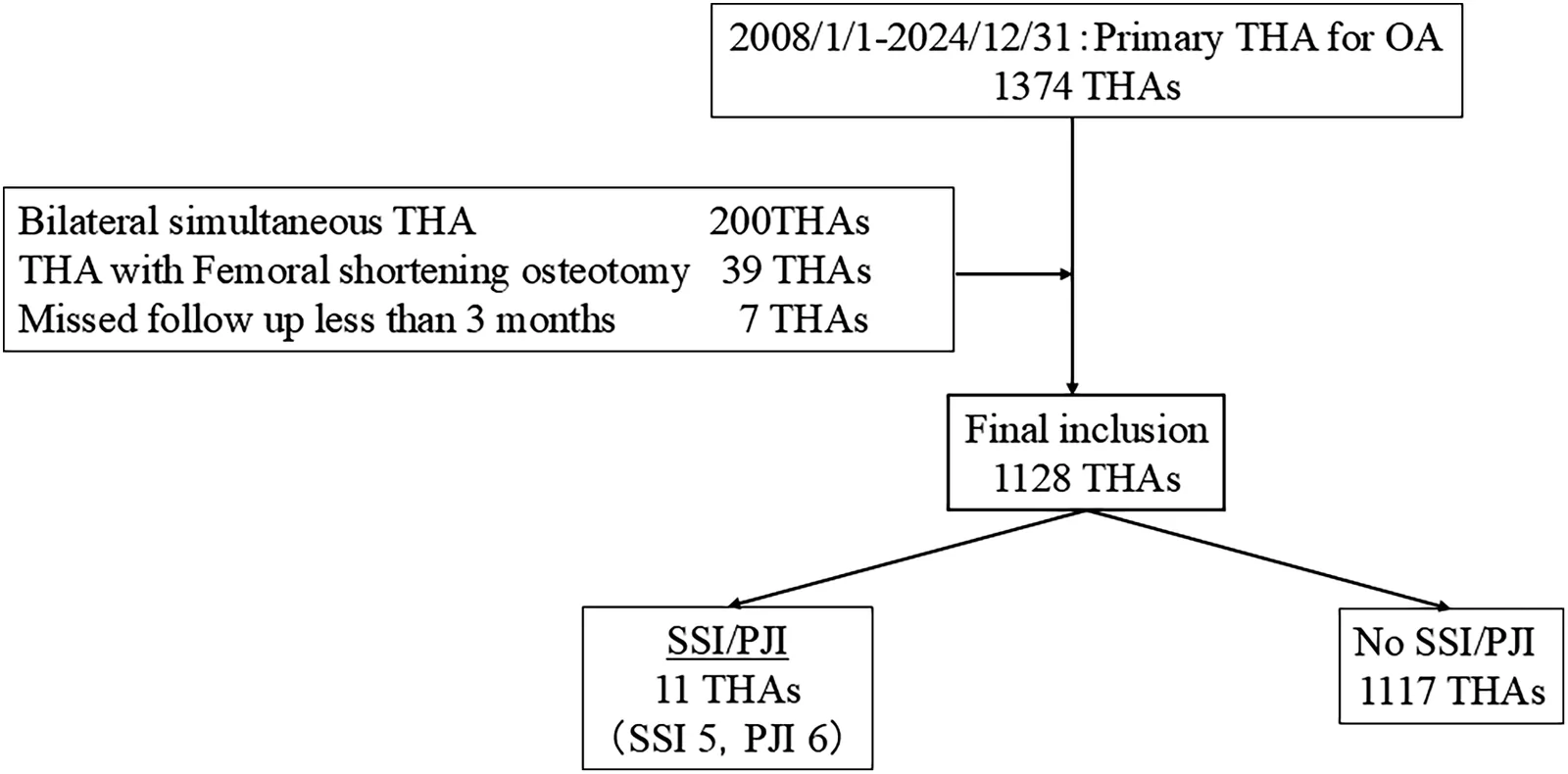
The flowchart of patient selection.

Regarding the grouping, 104 hips were included in the ASA-PS 1 group, 932 hips in the ASA-PS 2 group, and 92 hips in the ASA-PS 3 group. SSI/PJI occurred in no hips in the ASA-PS 1 group, eight hips (0.8%) in the ASA-PS 2 group, and three hips (3.3%) in the ASA-PS 3 group. No significant differences were observed in the mean age, sex, or age-adjusted CCI among the three groups. No patient had DM in the ASA-PS 1 group. In the ASA-PS 2 group, there was a significant difference in the number of patients who had DM between the two subgroups (PI and Saline), but there was no significant difference in the preoperative HbA1c levels. In the ASA-PS 3 group, there was no significant difference in the number of patients who had DM and preoperative HbA1c levels between the two subgroups.

No incidence of SSI/PJI was observed in the ASA-PS 1 group. In the ASA-PS 2 group, the PI subgroup had a significantly lower SSI/PJI rate (*P* = 0.04), although there was a significant difference in the number of patients who had DM between the two subgroups. Meanwhile, in the ASA-PS 3 group, the PI subgroup showed no significant reduction in SSI/PJI rate (*P* = 0.55) (Tables 1–3). OR revealed that diluted PI irrigation significantly reduced SSI/PJI incidence (OR, 0.23; 95% confidence interval [CI], 0.06–0.91; *P* = 0.03) in the ASA-PS 2 group (Table 4).

**Table 1:** Demographic data of enrolled patients for ASA-PS1.

	Saline (*n* = 36)	Iodine (*n* = 68)	*P*-value
Age	54.3 ± 8.5	56.9 ± 7.2	0.14
Male/Female	1/35	5/63	0.66
BMI	21.8 ± 2.8	21.3 ± 2.6	0.10
Age adjusted CCI			0.40
0	2 (5.5%)	10 (14.7%)	
1	10 (27.8%)	0	
2	13 (36.1%)	31 (45.6%)	
3	9 (25.0%)	19 (27.9%)	
4≧	2 (5.5%)	8 (11.8%)	
SSI/PJI	0	0	1.00

Age and BMI are shown in mean ± S.D. BMI, body mass index; CCI, Charlson comorbidity index; SSI, surgical site infection; PJI, periprosthetic joint infection.

**Table 2:** Demographic data of enrolled patients for ASA-PS2.

	Saline (*n* = 174)	Iodine (*n* = 758)	*P*-value
Age	62.9 ± 9.7	64.8 ± 9.8	0.21
Male/Female	22/152	103/655	0.80
BMI	23.1 ± 3.4	23.7 ± 3.8	0.15
Age adjusted CCI			0.52
0	4 (2.3%)	9 (1.2%)	
1	9 (5.2%)	31 (4.1%)	
2	40 (23.0%)	130 (17.2%)	
3	53 (30.4%)	230 (30.3%)	
4	38 (21.8%)	209 (27.6%)	
5≧	30 (17.2%)	149 (19.6%)	
DM	6 (3.4%)	82 (10.8%)	*<0.01*
HbA1c	6.4 ± 0.8	6.5 ± 0.7	0.90
SSI/PJI	4 (2.3%)	4 (0.5%)	*0.04*

Age, BMI, and HbA1c are shown in mean ± S.D.

BMI, body mass index; CCI, Charlson comorbidity index; SSI, surgical site infection; PJI, periprosthetic joint infection. Significance was defined as *P* < 0.05.

**Table 3:** Demographic data of enrolled patients for ASA-PS3.

	Saline (*n* = 28)	Iodine (*n* = 64)	*P*-value
Age	68.1 ± 10.6	66.0 ± 12.3	0.41
Male/Female	4/24	16/52	0.41
BMI	25.1 ± 5.2	27.2 ± 7.2	0.11
Age adjusted CCI			0.30
1–2	2 (7.1%)	7 (10.9%)	
3	1 (3.6%)	9 (14.0%)	
4	5 (17.9%)	13 (20.3%)	
5	10 (35.7%)	20 (31.4%)	
6≧	10 (35.7%)	15 (23.4%)	
DM	9 (32.1%)	13 (20.3%)	0.28
HbA1c	7.3 ± 0.9	7.0 ± 0.9	0.32
SSI/PJI	0	3 (4.7%)	0.55

Age, BMI, and HbA1c are shown in mean ± S.D. BMI, body mass index; CCI, Charlson comorbidity index; SSI, surgical site infection; PJI, periprosthetic joint infection.

**Table 4:** The odds ratio (OR) in the incidence of SSI/PJI.

	OR (95%CI)	*P*-value
ASA-PS 2	0.22 (0.05–0.91)	*0.03*

OR, Odds ratio; CI, confidence interval; ASA-PS, American Society of Anesthesiologists Physical Status. Significance was defined as *P* < 0.05.

## Discussion

It is essential to mitigate the incidence of SSI/PJI and implement intraoperative preventive measures for SSI/PJI according to international guidelines and findings from recent systematic reviews [5–7]. Of these measures, joint irrigation with antiseptic solutions, including chlorhexidine and PI solutions (but not antibiotics), is cost-effective and widely applicable [13]. Although recent systematic reviews and meta-analyses have reported the importance of intraoperative diluted PI lavage for preventing SSI/PJI after orthopedic surgery, no study has investigated the association between patient risk stratification for SSI/PJI and the use of diluted PI lavage in primary THA. Therefore, findings from our study may be used to guide patient stratification for diluted PI lavage to mitigate SSI/PJI after primary THA.

Previous studies have investigated the clinical effectiveness of diluted PI lavage for the prevention of SSI/PJI in primary total hip and knee arthroplasty. Shohat et al. investigated the efficacy of pre-closure diluted PI irrigation in a large single-center retrospective cohort. This study included 31,331 patients, of whom 8659 patients received 0.3% diluted PI irrigation, whereas 22,672 patients received saline irrigation prior to wound closure. Findings from this study showed a significant decrease in the incidence of PJI with increasing use of diluted PI irrigation (Adjusted OR, 0.397; 95% CI, 0.283–0.556; *P* < 0.001) [14]. Muwanis et al. also investigated the clinical impact of 0.325% diluted povidone irrigation prior to wound closure and its effect on postoperative acute infection rate after primary total hip and knee arthroplasty. This study showed that using diluted povidone irrigation led to a significant reduction in the incidence of SSI/PJI (OR, 0.45; 95% CI, 0.22–0.89; *P* < 0.05) [15]. Furthermore, a recent systematic review and meta-analysis by Machinski et al. analyzed data from 67,742 patients across 11 primary studies. The review reported that diluted PI irrigation significantly reduced the risk of PJI compared to saline irrigation in total joint arthroplasty (OR, 0.60; 95% CI, 0.37–0.95; *P* = 0.029) [16].

However, in clinical situations, the risk of SSI/PJI varies across patients, and there is a lack of consensus regarding the effectiveness of diluted PI irrigation even in patients at high risk of developing SSI/PJI. Some previous studies have indicated no significant reduction in SSI/PJI incidence with diluted PI irrigation after total joint arthroplasty [17, 18]. Therefore, we investigated the usefulness of patient stratification for diluted PI lavage to prevent SSI/PJI after primary THA. We found that 0.2% diluted PI lavage led to a significant reduction of SSI/PJI incidence in the ASA-PS 2 group; however, it did not have any significant effect on SSI/PJI incidence in the ASA-PS 3 group. Hence, diluted PI lavage could be useful for reducing the risk of SSI/PJI, as reported in previous studies. However, its effect may not be significant when the patient’s risk for developing SSI/PJI after primary THA is relatively high. Furthermore, our results may suggest that additional intraoperative measures are needed to mitigate SSI/PJI in patients at high risk of developing SSI/PJI after primary THA. Previous studies have investigated the usefulness of topical vancomycin powder combined with diluted PI irrigation [19, 20]. Meanwhile, a recent multicenter randomized controlled study by Sava et al. investigated the efficacy of local vancomycin powder, diluted PI lavage, or the vancomycin-PI combined protocol using normal saline irrigation as a reference. This study showed that there was no significant difference in the 3-month infection rates across all groups. The study concluded that the use of PI and vancomycin powder in high-risk patients can be left to the surgeon’s or hospital’s discretion [21]. Therefore, there is no consensus on the use of topical vancomycin powder combined with diluted PI irrigation to reduce SSI/PJI occurrence in high-risk patients owing to concerns regarding clinical effectiveness and potential for local antimicrobial resistance. In such situations, antimicrobial-coated implants may be a useful tool for mitigating SSI/PJI risk, as they are associated with fewer concerns regarding local antimicrobial resistance. However, further accumulation of clinical results is needed [22–24].

Our study had some limitations. This study was retrospective, and the accuracy of the collected data depended on the completeness of information recorded in the electronic medical database. Additionally, this study was conducted at a single institution. A power calculation (α = 0.05; power = 80%) for SSI/PJI after primary THA indicated that, at a 1% incidence rate, a cohort of 3353 patients is required. Consequently, our study is underpowered and cannot conclusively clarify the influence of diluted PI lavage on risk-adjusted SSI/PJI. Our study is limited by the very small number of SSI/PJI events and the long inclusion period, both of which substantially reduce the robustness of the findings. These factors also limit our ability to attribute differences in infection risk specifically to PI lavage, and the results should therefore be interpreted as exploratory. Even so, our findings add value because, to our knowledge, no study has comprehensively characterized how patient risk stratification relates to SSI/PJI incidence following primary THA. We intend to mitigate the constraints of this single-center analysis through a forthcoming multicenter study. Furthermore, owing to the small number of patients in the ASA-PS 3 group, it cannot be definitively stated that the lack of statistical significance indicates a clinically meaningful difference. Therefore, we plan to conduct a multicenter study to overcome this concern. Although irrigation was generally performed at approximately 30-min intervals, the exact timing varied depending on intraoperative circumstances; therefore, the reproducibility of irrigation timing was not absolute. The concentration of PI and the amount of irrigation may have affected the clinical outcomes in this study. However, as we mentioned above, the appropriate dilution concentration of PI and the appropriate amount of diluted PI irrigation were unknown; many previous studies used dilution concentrations of 0.13–0.35%. Accordingly, there is a need for standardized and well-powered randomized trials to identify optimal irrigation protocols. Finally, combining superficial SSI and early PJI into a single endpoint is a limitation of this study, as these complications differ in severity and pathophysiology. We combined them because both represent early postoperative wound-related infections that may be influenced by intraoperative antiseptic irrigation. In addition, our analysis focused only on superficial SSI and early PJI within 90 days. We did not evaluate late PJI; therefore, the long-term impact of diluted PI lavage remains unknown. Long-term follow-up studies on diluted PI lavage are needed.

In conclusion, this study showed that diluted PI irrigation may contribute to a significant reduction in the incidence of SSI/PJI after primary THA in patients with ASA-PS class 2. However, the incidence of SSI/PJI may not be significantly reduced in high-risk patients with ASA-PS class 3. This may suggest the need for additional effective intraoperative prophylaxis, such as antimicrobial-coated implants.

## Data Availability

The datasets generated and/or analyzed during the current study are available from the corresponding author on reasonable request.
